# Supplementary material for: Kidney Allograft Function Is a Confounder of Urine Metabolite Profiles in Kidney Allograft Recipients
Source: Metabolites. 2021 Aug 11;11(8):533. doi: 10.3390/metabo11080533 (PMC8399888; doi:10.3390/metabo11080533)
Supplement: Supplementary file 1 [file metabolites-11-00533-s001.zip › metabolites-1281040-supple/Table S1.pdf]

Table S1. Characteristics of Kidney Transplant Recipients at Baseline

Recipient Characteristics	Study Cohort	ACR Biopsy Group	AMR Biopsy Group	Mixed Rejection Biopsy Group	PVAN Biopsy Group	ATI Biopsy Group	Normal Biopsy Group
Patient Number[1], N	153	22	16	14	32	49	29
Biopsy Number, N	192	22	16	14	36	51	53
Sex, N (%)							
Male	99 (64.7)	15 (68.2)	8 (50)	10 (71.4)	21 (65.6)	34 (69.4)	18 (62.1)
Female	54 (35.3)	7 (31.8)	8 (50)	4 (28.6)	11 (34.4)	15 (30.6)	11 (37.9)
Race, N (%)							
White	69 (45.1)	13 (59.1)	7 (43.8)	4(28.6)	14 (43.8)	25 (51.0)	11 (37.9)
Black	47 (30.7)	8 (36.4)	4 (25)	6 (42.9)	11 (34.4)	12 (24.5)	9 (31.0)
Hispanic	19 (12.4)	0 (0)	2 (12.5)	3 (21.4)	4 (12.5)	4 (8.2)	7 (24.1)
Asian	12 (7.8)	1 (4.6)	3 (18.8)	1 (7.1)	1 (3.1)	5 (10.2)	1 (3.5
Other	6 (3.9)	0 (0)	0 (0)	0 (0)	2 (6.3)	3 (6.1)	1 (3.5)
Cause of ESRD, N (%)							
Diabetes	44 (28.8)	7 (31.8)	3 (18.8)	4 (28.6)	9 (28.1)	16 (32.7)	7 (24.1)
Hypertension	26 (17)	4 (18.2)	2 (12.5)	5 (35.7)	7 (21.9)	7 (14.3)	2 (6.9)
Glomerulonephritis	27 (17.7)	3 (13.6)	5 (31.2)	0 (0)	6 (18.8)	6 (12.2)	7 (24.1)
Polycystic Kidney Disease	17 (11.1)	2 (9.1)	2 (12.5)	0 (0)	3 (9.4)	5 (10.2)	7 (24.1)
Lupus	12 (7.8)	2 (9.1)	0 (0)	2 (14.3)	2 (6.3)	5(10.2)	2 (6.9)
Other	27(17.7)	4 (18.2)	4 (25)	3 (21.4)	5 (15.6)	10 (20.4)	4 (13.8)
Prior Transplant History, N (%)	24 (15.7)	5 (22.7)	5 (31.3)	4 (28.5)	4 (12.5)	7 (14.3)	1 (3.5)
Type of Donor, N (%)							
Deceased	80 (52.3)	13 (59.1)	6 (37.5)	7 (50)	20 (62.5)	26 (53.1)	13 (44.8)
Living	73 (47.7)	9 (40.9)	10 (62.5)	7 (50)	12 (37.5)	23 (46.9)	16 (55.2)

HLA Mismatch (A,B,DR), median	5	5	4	5	5	4	5
PRA[2] at Transplant, mean (SD)	15 (31)	19 (35)	35 (43)	16 (29)	7 (20)	12 (29)	14 (30)
HLA-DSA[3] at Transplant, N (%)	46 /148 (33)	5/22 (23)	10/16 (63)	5/14 (35.7)	4/30 (13.3)	15/46 (32.6)	11/28 (37.9)
Class I DSA only	11 (7.4)	1 (4.6)	1 (6.3)	0 (0)	3 (10)	2 (4.4)	5 (17.2)
Class DSA II only	21 (14.2)	2 (9.1)	4 (25)	1 (7.1)	0 (0)	10 (21.7)	6(20.7)
Class I and II DSA	14 (9.5)	2 (9.1)	5 (31.2)	4 (28.6)	1 (3.3)	3 (6.5)	0 (0)
Induction Therapy, N (%)							
Thymoglobulin	126(82.4)	15 (68.2)	13 (81.2)	10 (71.4)	29 (90.6)	39 (79.6)	29 (100)
IL-2 Receptor Antibody	23(15)	5 (22.7)	3 (18.8)	3 (21.4)	3 (9.4)	9 (18.4)	0 (0)
Other	4 (2.6)	2 (9.1)	0 (0)	1 (7.2)	0 (0)	1 (2.0)	0 (0)
Steroid Maintenance Therapy, N (%)	48 (31.4)	10 (45.5)	6 (37.5)	6 (42.9)	6 (18.8)	19 (38.8)	2 (6.9

ESRD, End Stage Renal Disease; HLA, Human Leukocyte Antigen; PRA, Panel Reactive Antibodies; PRA, Panel Reactive Antibodies; DSA, Donor Specific Antibodies to Donor HLA;

[1]Total number of patients studied was 153 with 27 patients providing more than one sample in the data set. Nine patients contributed samples to more than one group - two patients from Mixed Rejection group, two patients from ATI group and 5 patients from Normal group.

[2]PRA value was calculated using complement dependent cytotoxicity in 28% of the cohort and by single antigen bead assay on the Luminex platform in 72%.

[3]Presence of DSA was determined using solid phase PRA beads on the Luminex platform in 38 patients and using single antigen bead assay on the Luminex platform in 110 patients and not determined in 5 patients.
