# Supplementary material for: Kidney Allograft Function Is a Confounder of Urine Metabolite Profiles in Kidney Allograft Recipients
Source: Metabolites. 2021 Aug 11;11(8):533. doi: 10.3390/metabo11080533 (PMC8399888; doi:10.3390/metabo11080533)
Supplement: Supplementary file 1 [file metabolites-11-00533-s001.zip › metabolites-1281040-supple/Table S2.pdf]

Table S2. Kidney Allograft Biopsy Associated Parameters and Banff Histology Scores

Variables	ACR Biopsy Group	AMR Biopsy Group	Mixed Rejection Biopsy Group	PVAN Biopsy Group	ATI Biopsy Group	Normal Biopsy Group	P^1
Biopsies, N	22	16	14	36	51	53	
Age at biopsy, Years, Mean (SD)	50.5 (13.9)	41.8 (12.6)	40.9 (14)	56.9 (11.8)	51.2 (14.5)	53.7 (10)	
Biopy Type, N (%)							
For-Cause	22 (100)	15 (93.8)	14 (100)	36 (100)	50 (98)	0 (0)	
Surveillance	0 (0)	1 (6.2)	0 (0)	0 ()0	1 (2)	53 (100)	
Time Since Transplantation to Biopsy, Months							
Mean (SD)	25.8 (43.2)	16.1 (16.5)	36.9 (43.3)	17 (15.6)	10.4(18.6)	16.1 (13.1)	<0.001
Median	7.1	16.4	23.3	11.2	2.8	15.2	
Min, Max	0.2, 162	0.4, 59	0.13, 146	2.7, 70	0.2, 109.6	2.8, 42.8	
Serum Creatinine (mg/dL) at the Time of Biopsy							
Mean (SD)	5.1 (5.7)	2.2 (0.9)	5.8 (4.6)	2.5 (1.0)	3.4 (1.9)	1.4 (0.35)	<0.001
Median	2.9	2.0	4.4	2.2	2.8	1.4	
Min, Max	1.2, 23.9	1.2, 3.8	2.4, 20.2	1.1, 4.9	0.98, 8.5	0.75, 2.2	
Biopsy Associated DSA[2]							
No DSA, N (%)	12 (55)	0 (0)	0 (0)	19 (52.8)	30 (58.8)	39 (73.6)	<0.001
Class I DSA only	4 (18.2)	0 (0)	0 (0)	5 (13.9)	4 (7.8)	4 (7.6)	
Class II DSA only	3 (13.6)	6 (37.5)	4 (28.6)	4 (11.1)	10 (19.6)	9 (17)	

Class I and II DSA	1 (4.6)	10 (62.5)	10 (71.4)	2 (5.6)	1 (2)	1 (1.9)	
Not Determined (at biopsy)	2(9)	0 (0)	0 (0)	6 (16.7)	6 (11.8)	0 (0)	
C4d, Positive[3] N (%)	0 (0)	15 (93.8)	11 (78.6)	0 (0)	2 (3.9)	0 (0)	<0.001
C4d, Negative, N (%)	22 (100)	1 (6.25)	3 (21.4)	35(97.2)	49 (96.1)	51 (96.23)	<0.001
C4d Not Performed, N (%)	0 (0)	0 (0)	0 (0)	1 (2.8)	0 (0)	2 (3.8)	
Banff Scores[3], Mean (SD)							
Tubulitis (t score),	2.5 (0.51)	0.50 (0.63)	2.8 (0.43)	1.94 (1.1)	0 (0)	0 (0)	<0.001
Interstitial Inflammation (i)	2.6 (0.50)	0.81 (0.75)	2.6 (0.51)	2.2 (0.92)	0.10 (0.30)	0.02 (0.14)	<0.001
Glomerulitis (g)	0.68 (0.65)	1.25 (0.93)	1.3 (1.2)	0.31 (0.79)	0.04 (0.20)	0 (0)	<0.001
Peritubular capillaritis (ptc)	1.64 (0.9)	2.5 (0.52)	2.6 (0.84)	0.92 (0.87)	0.16 (0.51)	0.02 (0.14)	<0.001
Interstitial Fibrosis/ Tubular Atrophy (IFTA)	0.91 (1.1)	0.44 (0.82)	1.6 (1.0)	1.36 (0.99)	0.55 (0.67)	0.85 (0.91)	0.09

[1]P values were calculated for group comparisons using Chi-square test for categorical variables and one-way analysis of variance for continuous variables and semi-quantitative scores.
[2]Presence of circulating antibodies to donor HLA (DSA) in serum collected within 30 days of the biopsy procedure and prior to anti-rejection treatment treatment was analyzed using single antigen bead (SAB0 LABScreen LS1A04 and LS2A01 assays (One Lambda, Inc., Canoga Park, CA) on the Luminex platform; SAB Mean Fluorescent Intensity (MFI) value >1000 was classified as a positive result;
[3]Intrgraft deposition of C4d was deterimed using Indirect immunofluorescence for complement factor 4 degradation (C4d) product was performed on cryosections using a monoclonal anti-C4d antibody (Quidel, Santa Clara, CA).
[4]Biopsies were classified using the Banff 2017 diagnostic criteria and the Banff scores of individual lesion (e.g., Tubilitis (t) score= 0, 1, 2 or 3) were used to calculated the Median (SD) scores.
